# Genetic profiling of chromosome 1 in breast cancer: mapping of regions of gains and losses and identification of candidate genes on 1q

**DOI:** 10.1038/sj.bjc.6603433

**Published:** 2006-10-24

**Authors:** B Orsetti, M Nugoli, N Cervera, L Lasorsa, P Chuchana, C Rougé, L Ursule, C Nguyen, F Bibeau, C Rodriguez, C Theillet

**Affiliations:** 1 Génotypes et Phénotypes Tumoraux, EMI229 INSERM/Université Montpellier I, Centre de Recherche, CRLC Val D'Aurelle-Paul Lamarque, Montpellier cedex 5 34298, France; 2 ERM 206 INSERM/Université Aix-Marseille2, Parc Scientifique de Luminy Marseille, France; 3 Department of Pathology, CRLC Val D'Aurelle-Paul Lamarque, Montpellier, France

**Keywords:** array-CGH, amplicon, oncogene, profiling

## Abstract

Chromosome 1 is involved in quantitative anomalies in 50–60% of breast tumours. However, the structure of these anomalies and the identity of the affected genes remain to be determined. To characterise these anomalies and define their consequences on gene expression, we undertook a study combining array-CGH analysis and expression profiling using specialised arrays. Array-CGH data showed that 1p was predominantly involved in losses and 1q almost exclusively in gains. Noticeably, high magnitude amplification was infrequent. In an attempt to fine map regions of copy number changes, we defined 19 shortest regions of overlap (SROs) for gains (one at 1p and 18 at 1q) and of 20 SROs for losses (all at 1p). These SROs, whose sizes ranged from 170 kb to 3.2 Mb, represented the smallest genomic intervals possible based on the resolution of our array. The elevated incidence of gains at 1q, added to the well-established concordance between DNA copy increase and augmented RNA expression, made us focus on gene expression changes at this chromosomal arm. To identify candidate oncogenes, we studied the RNA expression profiles of 307 genes located at 1q using a home-made built cDNA array. We identified 30 candidate genes showing significant overexpression correlated to copy number increase. In order to substantiate their involvement, RNA expression levels of these candidate genes were measured by quantitative (Q)-RT–PCR in a panel of 25 breast cancer cell lines previously typed by array-CGH. Q–PCR showed that 11 genes were significantly overexpressed in the presence of a genomic gain in these cell lines, and 20 overexpressed when compared to normal breast.

Chromosome 1 is recurrently altered in a number of human malignancies. In solid tumours, structural aberrations include several recurrent chromosomal translocation sites, as well as frequent gains or losses involving either chromosomal arm (Struski *et al*, 2002; Teixeira *et al*, 2002). In breast cancer, chromosome 1 is the site of rare stereotypic rearrangements; isochromosome i(1)(q10), and der(1;16)(q10;p10) (Tsarouha *et al*, 1999). More significantly, it has been shown, by either LOH or CGH work, to be frequently involved in copy number changes (CNCs) (Kerangueven *et al*, 1997; Osborne and Hamshere, 2000). Fifty to 60% of breast tumours analysed by CGH presented gains at 1q, whereas the short arm showed predominantly losses, except the 1p31–p32 region which presented occasional gains (Courjal and Theillet, 1997; Tirkkonen *et al*, 1998). Gains at 1q frequently affect the whole arm; however, a number of tumours or cell lines exhibit interstitial gains sometimes reduced to a chromosomal band or sub-band (Courjal and Theillet, 1997; Larramendy *et al*, 2000). These data suggesting the existence at 1q of several regions of gains were thus concordant with LOH studies, indicating the occurrence of at least four regions of allelic imbalance in breast tumours (Kerangueven *et al*, 1997). Because gains at 1q were observed both in low- and high-grade breast tumours, its implication in early stages of disease development has been shown (Tirkkonen *et al*, 1998; Cummings *et al*, 2000). Recent data using BAC-based array-CGH on independent sets of breast tumours have confirmed the frequent nature of gains on chromosome 1, as well as the existence of multiple cores of amplification (Stange *et al*, 2006).

Altogether, these data suggested the presence of several important cancer genes on chromosome 1. Several known oncogenes (*NRAS, JUN, MYCL, TAL1, BLYM, LCK*) map on chromosome 1q, but their implication in breast cancer has remained elusive, whereas genes like *MUC*1 and *PLU-*1/*JARIB1* were proposed as candidates (Bieche *et al*, 1997; Lu *et al*, 1999). However, it seems clear that most genes involved remain to be identified. This notion was reinforced by recent expression profiling studies in breast tumours that showed that 25 genes located on the long arm of chromosome 1 showed increased expression levels in conjunction to DNA copy number increase (CNI) (Hyman *et al*, 2002).

Our goal in this work was to determine more precisely the boundaries of regions of chromosome 1 showing CNCs in breast tumours and gain insight on genes involved. To achieve this, we built a genomic array covering both arms of chromosome 1 at an average density of one BAC clone/0.85 Mb and analysed 30 breast cancer cell lines and 30 primary breast tumours by array-CGH. Based on the array-CGH profile, we defined shortest regions of overlap (SROs) of copy number gain or loss. A total of 20 regions of loss, all located at chromosome 1p, and 19 regions of gain, one at 1p and 18 at 1q, were defined. Because gains at 1q were found in over 60% of the analysed samples and increased copy number are clearly related to augmented gene expression, we focused our expression study on the identification of candidate genes at 1q. To this aim, we studied expression profiles of 307 known genes located on the long arm of chromosome 1. Using a supervised analysis method, we selected 30 genes showing significantly increased RNA expression in relation to genomic gains. RNA expression levels of 28 out of 30 genes were verified by quantitative (Q)–RT–PCR and the overexpression in relation to gains was confirmed for 11 out of 28 genes, whereas 20 out of 28 showed overexpression compared to normal breast.

## MATERIALS AND METHODS

### Tumours and blood samples

Thirty breast tumours were obtained from the Pathology Department at the Val d'Aurelle Cancer Center of Montpellier (France). Tumour biopsies were snap-frozen in liquid nitrogen upon surgical removal and stored at −80°C until DNA and RNA extraction. Tumour cohort was composed of 63.7% invasive ductal carcinoma, 18% invasive lobular carcinoma, 15% invasive adenocarcinoma of unspecified type and 3.3% other types of carcinomas of the breast. The mean age of patients was 58 years. Tumours were mostly grade 2 and 3 (46.7 and 29.2%, respectively), whereas 13.9% were grade 1 and 10% were uninformed.

### Cell lines and tumours

Breast cancer cell lines used in this study included BRCAMZ01, MDAMB175, MDAMB453 (D Birnbaum, INSERM U119, Marseille, France), CAL51, MDAMB435, SKBR7, ZR7530 (P Edwards, Department of Pathology, Cambridge, UK), BT474, MCF7Rich (F Vignon, INSERM U540, Montpellier, France), HS578T, MDAMB436, (A Puisieux, INSERM U590, Lyon, France), SUM149, SUM185, SUM52 (S Ethier, University of Michigan, Ann Arbor, MI, USA), EFM19, (DSMZ, Braunschweig – Germany), BT20, BT483, HCC1187, HCC1395, HCC1428, HCC1937, HCC1954, HCC2218, MDAMB157, MDAMB361, MDAMB468, SKBR3, T47D, UACC812 and ZR751 (ATCC, American Type Culture Collection, Manassas, VA, USA). All cell lines were cultured as recommended by suppliers.

### Genomic arrays

We built a genomic array covering chromosomes 1, 8 and 17. Coverage of chromosomes 8 and 17 has been described by Orsetti *et al* (2004) and Gelsi-Boyer *et al* (2005). Chromosome 1 was covered by 257 BAC clones selected as follows: 225 BAC clones from the Barbara Trask collection (CHORI) http://www.ncbi.nlm.nih.gov/genome/cyto/hbrc.shtml and 32 clones selected according to their cytogenetic position and content in genetic markers. Clones were arranged according to the human genome freeze of April 2003. This resulted in an average density of one clone/0.85 Mb±0.95 Mb. However, clone distribution was uneven and thus could produce local variations in resolution (a complete list of BAC clones with precise coordinates is available in Supplementary Table S1).

Arrays were produced according to the following procedure. BAC, PAC and Cosmid DNA were isolated using Nucleobond BAC100 from Macherey-Nagel (Hoerdt, France). Probe DNA to be spotted was prepared by DOP-PCR amplification on 10 ng of BAC matrix DNA in a final reaction volume of 100 *μ*l. Primer sequences and DOP-PCR protocol used are available on the Sanger Center web site (http://www.sanger.ac.uk/HGP/methods/cytogenetics/DOPPCR.shtml) (Orsetti *et al*, 2004). We performed this with slight modifications: the second round DOP-PCR primer was not aminolinked. Purification of PCR products was carried out using Nucleofast 96 PCR plates (Macherey-Nagel, Hoerdt, France). Purified PCR products were re-suspended in dd H_2_O at 2 *μ*g *μ*l^−1^. An aliquot was run on an agarose gel in order to ascertain even distribution of the product in all the wells. Prior spotting products were diluted 1 : 1 in spotting solution (GE-Healthcare, Orsay, France) and spotted in quadriplicate onto Corning GapsII slides (Schiphol-Rijk, The Netherlands) using a Lucidea array spotter IV (Amersham Biosciences, Orsay, France).

### Array-CGH probe labelling, hybridisation, image capture and data analysis

Genomic DNA was digested by *Nde*II according to the supplier's recommendations (Roche Diagnostics, Meylan, France). Three hundred nanograms of digested genomic DNA was labelled by random priming in a 50 *μ*l reaction containing 0.02 mM dATP, 0.02 mM dGTP, 0.02 mM dTTP, 0.05 mM dCTP, 0.04 mM Cy3-dCTP or Cy5-dCTP, 25 U of Klenow Fragment (50 U *μ*l^−1^, New England Biolabs, Ozyme, Saint Quentin Yvelines, France), 10 mM -mercaptoethanol, 5 mM MgCl_2_, 50 mM Tris-HCL (pH 6.8) and 300 *μ*g ml^−1^ random octamers. The reaction was incubated at 37°C for 20 h and stopped by adding 2.5 *μ*l EDTA 0.5 M pH 8. The reaction product size was about 100 bp. We purified labelled products using microcon 30 filters (Amicon, Millipore, Molsheim, France). Abundance of the labelled DNA was checked using a spectrophotometer and incorporation of dyes was calculated using Molecular Probes software (http://www.probes.com/resources/calc/basedyeratio.html). A mix of 700 pmol Cy5- and 700 pmol Cy3-labelled probes was ethanol-precipitated in the presence of 250–300 *μ*g of human Cot-1 DNA (Roche Diagnostics, Meylan, France) and 100 *μ*g herring sperm DNA (Promega, Charbonnières, France). The pellet was dried and re-suspended in 110 *μ*l Hybrisol VII (Appligene Oncor, Qbiogen, Illkirch, France). The probes were denatured at 80°C for 10 min, and repetitive sequences were blocked by pre-annealing at 37°C for 30 min.

Slide processing was performed using a HS4800 hybridisation station (Tecan, Lyon, France). Slides were treated with a blocking buffer (5 × SSC, 0.2% SDS, 1% BSA) at 42°C for 30 min and washed three times at 42°C using 2 × SCC, 0.2% SDS. Pre-annealed probes were injected in the chambers and hybridisation took place at 37°C for 16 h with mild agitation. Post-hybridisation washes were as follows: three washes at 52.5°C in solution 1 (2 × SSC, 0.2% SDS), followed by three washes in solution 2 (0.5 × SSC, 0.2% SDS) and one wash in solution 3 (0.1 × SSC) at 52.5°C. To remove salt and detergent residues, a brief wash with dd water was performed at 37°C and slides were dried within the chambers by an injection of N_2_ at 30°C.

Arrays were scanned by a GenIII Array Scanner (Amersham Biosciences, Orsay, France). Images were analysed by ARRAY-VISION 6.0 software (Amersham Biosciences, Orsay, France). Spots were defined by use of the automatic grid feature of the software and manually adjusted when necessary. Fluorescence intensities of all spots were then calculated after subtraction of local background. These data were then analysed using a custom made MS-Excel VBA script. Cy3 and Cy5 global intensities were normalised with the entire set of spots on the array, Cy3/Cy5 ratios were calculated, each BAC clone was spotted in four replicates, the median values of replicate spots were calculated and these values were used to define the selection threshold for individual spots. Only replicates showing less than 15% of deviation from the median were kept and a clone was taken into consideration when at least three of four replicates showed values within the 15% deviation limit. Representation of profiles with log 2 ratios in *Y*-axis and Mb position of clones (http://genome.ucsc.edu, April 2003 freeze) along the chromosome in *X*-axis. For each sample, at least two experiments were performed (Cy3/Cy5 and Cy5/Cy3), and the final profile corresponds to the mean of two experiments.

### RNA expression profiling of chromosome 1q using cDNA arrays

Variations in gene expression levels were analysed by large-scale measurement with home-made cDNA mini-arrays (7.5 × 9 cm; 720 human genes; 11 genes cm^−2^) produced as described (Nugoli *et al*, 2003). More specifically, our mini-arrays comprised 319 ESTs corresponding to 307 known genes mapping at 1q (Supplementary Table S2). Selection of cDNA clones was performed according to information gathered (and crosschecked) from different web-based databases; Genemap: http://www.ncbi.nlm.nih.gov/genemap99/, Genecards: http://genecards.weizmann.ac.il/, Genelynx: http://www.genelynx.org/ or UCSC Genome Browser, release April 2003: http://genome.ucsc.edu/. Hybridisation signals were quantified using the HDG Analyzer software (Genomic Solutions, Ann Arbo, MI, USA) by integrating all spot pixel signal intensities and removing spot background values determined in the neighbouring area.

### Quantitative RT–PCR

RNAs from cell lines and normal breast samples used for real-time PCR were isolated using the RNeasy Minikit (Qiagen, France) in accordance with the supplier's conditions. 1 *μ*g of total RNA, treated beforehand with RNase-free DNase (Promega, France), was reverse-transcribed using the SuperScript II RT and 250 ng of random hexamers (Invitrogen, France). Q–PCR reactions were carried out in an ABI Prism 7000 instrument (Applied Biosystems, France) in a final volume of 15 *μ*l according to the supplier's recommendations using SYBR Green as a detector. Primers were as described in Supplementary Table S3 in the supplementary data. We designed the primers for 17 genes, with the assistance of the Primer Express software (Applied Biosystems, France), and for the remaining 14 we used the Quantitect Primer Assays from the Gene Globe database (Qiagen, France). *ESRRG* primers were as described by Ariazi *et al* (2002). Standard curves were determined for each gene analysed by the use of serial dilutions from the same pool of cDNAs. Relative quantities were calculated referring to these curves and relative expression levels of each target gene was normalised to 28S RNA.

### Identification of aberrantly expressed genes in regions of CNC

We applied a supervised analysis scheme to identify genes significantly correlated to CNCs. Sample selection was based on array-CGH profiles. For each consensus region, samples presenting at least 25% of the BACs included in the region with log 2 ratio exceeding 0.25 were considered as amplified. For each available gene at 1q, we computed a discriminating score (DS) by comparing expression levels between the subgroup of samples presenting amplification (subgroup 1) and the subgroup of samples without amplification (subgroup 2). Discriminating score (Golub *et al*, 1999) was defined as DS=(M1−M2)/(S1+S2), where M1 and S1 represent mean and the s.d. of expression levels of one gene in subgroup 1, M2 and S2 in subgroup 2. Confidence levels were calculated by performing 1200 iterative random permutations per gene as described previously (Bertucci *et al*, 2004). Significance threshold for expression differences was DS⩾0.32 corresponding to <0.01 false positive. For Q–PCR results, we applied a *t*-test analysis.

## RESULTS

### Patterns of gains and losses at chromosome 1 in breast cancer

**Figure 1:**
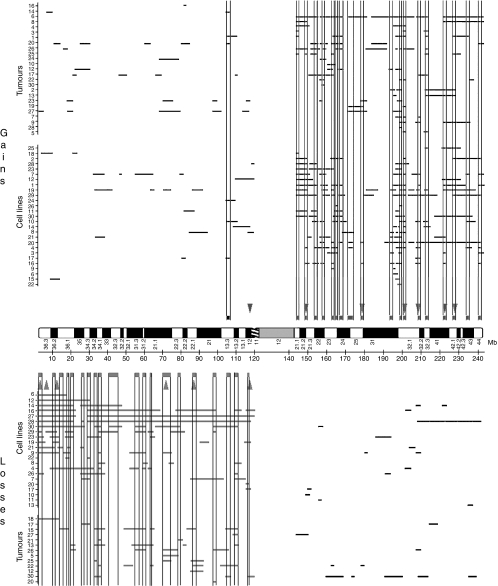
Profiles of gains and losses on chromosome 1 in breast cancer. Definition of SROs and events of high magnitude. Grey horizontal lines represent regions of gains (top) or losses (bottom) observed in each tumour or cell line (minimum two BACs involved with a log 2 ratio ⩾0.25 or ⩽−0.25). Shortest regions of overlap are indicated as bold grey bars with gains above the chromosome ideogram and losses below. Shortest regions of overlap correspond to the smallest overlap shared by at least six tumours or cell lines. Arrow heads indicate events of high magnitude, either peaks of amplification or loss. They corresponded to events with log 2 ratio >0.7 in at least three tumours or cell lines. Code for cell lines 1: BRCAMZ01, 2: BT20, 3: BT474, 4: BT483, 5: CAL51, 6: EFM19, 7: HCC1187, 8: HCC1395, 9: HCC1428, 10: HCC1937, 11: HCC1954, 12: HCC2218, 13: Hs578T, 14: MCF7Rich, 15: MDAMB157, 16: MDAMB175, 17: MDAMB361, 18: MDAMB435, 19: MDAMB436, 20: MDAMB453, 21: MDAMB468, 22: SKBR3, 23: SKBR7, 24: SUM52, 25: SUM149, 26: SUM185, 27: T47D, 28: UACC812, 29: ZR751 and 30: ZR7530. Code for primary tumours 1: VA1593, 2: VA4055, 3: VA4380, 4: VA4390, 5: VA4435, 6: VA4956, 7: VA5033, 8: VA5077, 9: VA5101, 10: VA5410, 11: VA5450, 12: VA6088, 13: VA6190, 14: VA6204, 15: VA6219, 16: VA6277, 17: VA6582, 18: VA6586, 19: VA6660, 20: VA7079, 21: VA7106, 22: VA7417, 23: VA6052, 24: VA6094, 25: VA6138, 26: VA6143, 27: VA6270, 28: VA6403, 29: VA6603 and 30: VA7072.

**Figure 2:**
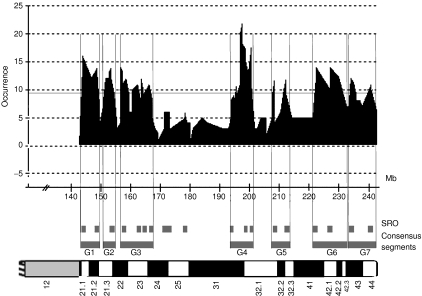
Definition of consensus regions of gain at 1q. Consensus regions were based on the curve of cumulated occurrence of gains (log 2.ratio ⩾0.25) at 1q in 30 cell lines and 30 primary tumours. Low points defined boundaries and high points possible cores. Only regions showing an occurrence exceeding the mean (9.0) were considered. Plots are based on the Mb positioning of the clones on the array. Hence, clones positioned close to each other may appear as merged. Consensus regions of gains were designated G1 through G7 and represented as bold grey lines. Short grey lines represent the position of SROs relative to that consensus regions.

We analysed genomic profiles of 30 primary tumours and 30 cancer cell lines by array-CGH using a home-built array covering chromosome 1 at an average density of one clone/0.85 Mb, with some local variations resulting in higher density locally at 1q. All cell lines studied, preselected on the basis of classical CGH profiles, presented gains and/or losses at either 1p or 1q. Array-CGH profiles were in good concordance with classical CGH data, confirming the prevalence of losses on the short arm combined with gains at 1q. However, in contrast to classical CGH data, gains encompassing the whole 1q were rare, with profiles typically showing multiple subregions of gains (Figure 1 and Supplementary Figure S1). Most prevalent gains were at 1q21–q22, 1q23–q24, 1q32 and 1q42–q44, whereas losses were noticeably rare on the long arm (Figures 1 and 2). On average, tumours and cell lines presented 1–3 regions of gains per sample (Figure 1).

Our aim was to define the cores of the different regions of CNC on chromosome 1 and thus, it was important to determine their boundaries. Correspondingly, we delineated the SROs involved in either gains or losses on the whole chromosome 1. We overlaid all the array-CGH profiles and searched for shortest overlaps shared by at least six independent tumours or cell lines. We defined 19 SROs of gains (one at 1p and 18 at 1q) and 20 SROs of losses (all at 1p) whose sizes ranged from 170 kb to 3.2 Mb (Figure 1). Precise locations and BAC content are described in Supplementary Data (Table S4). However, it must be pointed out that the actual sizes of these regions of overlap may change according to the resolution of the array used to define them.

Although, gains were generally of low-to-moderate level, high magnitude amplifications were observed. Similarly, we observed high magnitude losses (Figure 1). We were interested to see whether high magnitude amplifications occurred at recurrent sites and, accordingly, defined seven peaks of amplification, which all, except that at 1p12, matched with SROs (Figure 1). This discrepancy can be explained by the different criteria used to define peaks of amplification and SROs. Whereas SROs required to be shared by at least six samples to be retained, peaks of amplification needed to occur in at least three tumours or cell lines.

### Identification of candidate genes involved in CNCs at 1q

Because the relation between genomic gains and increased RNA expression is well established and linked to a selective advantage for cancer cells, we concentrated our efforts on the identification of the genes showing significantly increased expression levels as a consequence of gains at 1q. To this mean, we analysed RNA expression profiles of 307 genes located on chromosome 1q in 29 cell lines and 26 tumours using self-made cDNA arrays. We performed a supervised analysis aiming at selecting genes differentially expressed in tumours or cell lines presenting a gain. We formed groups of tumours and cell lines according to their ‘gain’ or ‘no gain’ status in each region. However, based on the 19 SROs, this resulted in a large number of subclasses whose samples were too small to reach statistical significance. To obviate this problem we defined larger regions of gains, designated consensus regions, which encompassed several SROs. To do this, we determined the occurrence curve for gains at each target clone at 1q. We reasoned that ruptures and low points in the curve represented the boundaries of the different regions (Figure 2). We retained only the events whose occurrence exceeded the mean (horizontal bar on Figure 2) and boundaries were defined by vertical lines tangential to the occurrence curve. Seven consensus regions of gains (G1 through G7), ranging from 3.6 to 11 Mb and encompassing two to three SROs on average were defined at 1q (Table 1 and Figure 2). Gains located between 170 and 180 Mb were not considered because their occurrence was below the threshold. Of the 307 genes studied, 178 genes were located within the consensus regions of gains defined at 1q. To identify genes whose expression levels were significantly modified in relation to CNC, we calculated the DS followed by 1200 random permutations (gain *vs* no gain) and our significance threshold for expression differences was DS⩾0.32 corresponding to <0.01 false positive. This resulted in the selection of 30 genes distributed in consensus regions G1 through G7 (Table 2). Interestingly, we noted that a number of the selected genes were located in close vicinity to each other suggesting the existence of local clusters, possibly related to the existence of core regions of gain.

### Candidate gene verification by Q–RT–PCR

In order to confirm expression profiling results, we measured the RNA expression levels of 28 out of 30 genes by Q–RT–PCR in 25 cell lines typed by array-CGH. The c1orf2 and *HNRPU* genes could not be studied because of unsuccessful primer design. In addition to the 28 genes selected from the cDNA array data, we studied the recently identified candidate oncogene *RAB25* (Cheng *et al*, 2004), which is located in consensus region G2, in close vicinity to two of our candidate genes, *MAPBPIP* and *CCT3* (Table 2). A *t*-test analysis revealed that only 5 out of 29 genes showed *P*-values =<0.05, indicative of significant expression differences in relation to gains. We reasoned that this may be owing to small sample size (we had to restrict our Q–PCR analysis to the 25 cell lines because tumour RNAs were no more available) and decided to consider genes with *P*-values =<0.1. This allowed us to pick out a total of 11 genes (Table 2). It was, however, noticeable that the *RAB25* gene was not selected in this test, whereas it was, when we compared mean expression levels in cancer cell lines to that in a series of five normal breast tissues expression (*t*-test *P*-value=0.002). We, thus, applied this test to the whole set of genes which revealed that 21 out of 29 genes were significantly overexpressed in cancer cell lines compared to normal breast.

## DISCUSSION

Chromosome 1 is a prevalent site of numerical anomalies combining losses on the short arm and gains on the long one in breast carcinomas (Courjal and Theillet, 1997; Teixeira *et al*, 2002). Gains at 1q are found in over 50% of breast tumours. Although being frequent in high-grade breast cancer, they have been related to ER-positive cancers (Rennstam *et al*, 2003; Loo *et al*, 2004) and have been suggested to occur early in the natural history of the disease (Buerger *et al*, 1999; Malamou-Mitsi *et al*, 1999). These particularities fostered our interest in characterising the genomic regions involved in CNCs and identifying genes at 1q whose expression was modified in relation to gains.

Array-CGH data presented here confirm chromosomal CGH results showing the duality on chromosome 1, with the short arm being mainly involved in losses and the long arm almost exclusively in gains. Our data clearly indicated the existence of multiple subregions of losses at 1p and of gains at 1q. In an attempt to define these subregions with greater precision and possibly delimitate their cores, we determined the SROs for gains (19 SROs) and losses (20 SROs) on chromosome 1, whose sizes ranged from 170 kb to over 3 Mb. Shortest regions of overlap were defined according to the classical LOH scheme, in order to narrow down genetic intervals encompassing candidate genes. Our data thus suggest that numerical anomalies at chromosome 1, be it losses or gains, are complex and involve a large number of subregions and possibly combinations of anomalies.

Although losses at 1p were observed in a sizeable portion of the tumours and cell lines, gains were notably prevalent. This was in full agreement with previous chromosomal CGH results by us and other groups (Courjal and Theillet, 1997; Tirkkonen *et al*, 1998; Malamou-Mitsi *et al*, 1999; Larramendy *et al*, 2000). Interestingly, gains at 1q were of low-to-moderate level with a lower prevalence of amplifications compared to other chromosomes. Furthermore, no sharp transitions were observed at the boundaries of amplification peaks at 1q, in contrast to chromosomes 8p or 17q, where such recurrent breakpoint sites were common (Orsetti *et al*, 2004; Gelsi-Boyer *et al*, 2005).

The relation between aberrant gene dosage and gene expression is well accepted and is best shown in case of CNI. The common nature of genomic gains (which include DNA amplification) in breast tumours indicates that it is an effective mechanism of positive genetic selection in cancer cells (Upender *et al*, 2004). By cDNA-array expression profiling, we identified 30 genes whose RNA expression was significantly increased in relation to genomic gains. Overexpression in the presence of genomic gain could be confirmed only for 11 genes by Q–RT–PCR. We suspect that these numbers may be related to the small size of our sample. We had to restrict our Q–PCR verification to 25 cell line RNAs, because tumour RNAs were no more available. We noted that 21 genes presented significant overexpression when compared to normal breast, suggesting the involvement of a larger number of genes within our original selection. It was interesting to see that, to the exception of *PLU-1/JARID1B* (Lu *et al*, 1999), all the genes identified in our study were newly proposed as candidate cancer genes. Furthermore, *MUC1* (Schroeder *et al*, 2004), a long known cell surface marker overexpressed in a sizeable fraction of breast tumours, and *KIF14* (Corson *et al*, 2005), a recently proposed candidate at 1q31, presented DSs below the threshold and were excluded from our selection. Genes selected in our study belong to rather diverse functional groups, of which three appeared prevalent. The first corresponded to a broad collection of positive regulators of cell proliferation. They include *PIP5K1A, MAPBPIP, RAB25A, PCTK3, RAB4* and *MPZL1*. The second was made of genes whose products were related to transcriptional regulation or chromatin remodelling such as *USF1, JARID1B, TBX19* or *CROC4*. The third included genes involved in cellular trafficking *VPS45A*, *ARF1*, *LYST, CCT3* or basic cellular metabolism *CA14*, *ALDH9A1*. Note that *RAB25* has also been related to the activation of protein trafficking between the membrane and the endoplasmic reticulum (Cheng *et al*, 2005). Similar functional groups have been observed in other selections of genes involved in genomic gains or amplifications, thus indicating the importance of activated transcription, increased signalling and protein trafficking or catabolism in cancer. However, 8 out of 24 overexpressed genes did not belong to any of the above-mentioned functional groups. Although two genes, *PDZK1* and *MLLT11,* were clearly relevant to cancer as both have been proposed as a candidate oncogene in diverse haematological malignancies (Busson-Le Coniat *et al*, 1999; Inoue *et al*, 2004; Tse *et al*, 2004), six were more difficult to relate to cancer. Three corresponded to genetic determinants of genetic syndromes (*MTMR, DISC1*, *MTX1)* and the three others bore functions with no obvious link to cancer *(NENF*, *ENSA, TARBP1).*

We were interested to verify the concordance between our analysis and the recently described ‘Transcriptome Correlation Map’ (Reyal *et al*, 2005), which defined groups of collinear genes showing coordinated expression. Their data set indicated 235 genes presenting a significant Transcriptome Correlation Score (TCS) at 1q, of which 147 mapped within the region of gains defined in our work, of which 72 (48%) were located in G1 and G2 (1q21 or 1q22). Genes within consensus regions of gains presented a significantly higher TCS, thus being in accordance with the existence of a link between increased expression and copy number gains at 1q. This was further corroborated by the fact, that 14 out of 30 (43%) genes selected by DS showed significant TCS, which is an increase compared to the 55 out of 178 (31%) genes common to both studies and located in the regions of gains. This suggested an enrichment of genes belonging to the transcriptome correlation map in our set of candidate genes at 1q and contrasted to our previous findings at 8p (Gelsi-Boyer *et al*, 2005).

Despite their frequent nature, numerical anomalies affecting chromosome 1 in breast and other cancers have drawn less attention than deserved. Most studies focussed on specific subregions or candidate genes. In this work, we characterised at high-resolution regions recurrently involved in copy number alterations on chromosome 1 in breast cancer and identified 24 candidate genes overexpressed in regions of gains at 1q. To our knowledge, this is the first study mapping at high-resolution regions of loss and gain on the whole length of chromosome 1 and proposing a series of candidate genes affected by CNCs. Further work will need to ascertain the true relevance to breast cancer of these candidate genes. This will require bioclinical and functional studies. Moreover, as our screen was based on a set of 307 known genes representing 40–50% of the genes assigned at 1q, our selection leaves way to the identification of additional candidate genes.

## Figures and Tables

**Table 1:** Description of consensus regions of gain at 1q

	**Consensus segments**	**Genomic positions**	**Size (bp)**	**Cytoband**	**BAC names**	**SRO included**	**Number of genes on our array**
G1	Start	143154718	5191576	1q21.1	CTD-2122l24	2-3	31
	End	148346294		1q21.3	RP11-74C1		
							
G2	Start	150842537	3669729	1q21.3	RP11-73C10	4	36
	End	154512266		1q23.1	RP11-91g5		
							
G3	Start	157448999	9571469	1q23.3	RP11-79m15	5-6-7-8	42
	End	167020468		1q24.2	RP11-184n12		
							
G4	Start	194594372	7404055	1q31.3	RP11-321M13	11-12-13	33
	End	201998427		1q32.1	CTD-2218h7		
							
G5	Start	208699401	5211392	1q32.3	RP11-216f1	14-15	8
	End	213910793		1q41	RP11-260a10		
							
G6	Start	223358648	11257879	1q42.12	CTD-2148o23	16-17	19
	End	234616527		1q43	RP11-80p14		
							
G7	Start	235845765	8332672	1q43	RP11-130i13	18-19	9
	End	244178437		1q44	RP11-172p12		

BAC=bacterial artificial chromosome; SRO=shortest region of overlap.

Consensus regions of gain were defined by the BAC bording them, Mb start corresponds to the 5′ end of the proximal BAC, Mb end to the 3′ end of the distal BAC.

**Table 2:** Gene expression analysis at 1q and correlation with copy number gain

**Consensus segment**	**Clone ID on the chip**	**Hugo gene symbol**	**Gene name**	**Localisation (start–end) (bp)**	**Cytoband**	**P-value 1**	**P-value 2**
G1	pdzk1	*PDZK1*	PDZ domain containing 1	143403500–143439848	1q21.1		0.015
G1	h2bfq	*HIST2H2BE*	Histone 2, H2be	146631105–146633327	1q21.2		
G1	cra	*MTMR11*	Myotubularin-related protein 11	146675639–146683822	1q21.2	0.09	0.011
G1	vps45b	*VPS45A*	Vacuolar protein sorting 45A	146814958–146892599	1q21.2	0.025	0.027
G1	ca14	*CA14*	Carbonic anhydrase XIV	147005313–147012571	1q21.2		0.019
G1	ensa	*ENSA*	Endosulfine alpha	147370158–147377163	1q21.3	0.05	0.025
G1	anxa9	*ANXA9*	Annexin A9	147729649–147743202	1q21.3		
G1	af1q	*MLLT11*	Myeloid/lymphoid or mixed-lineage leukaemia; translocated to, 11	147807778–147816066	1q21.3		0.045
G1	pip5k1a	*PIP5K1A*	Phosphatidylinositol-4-phosphate 5-kinase, type I, alpha	147897780–147948713	1q21.3	0.09	
							
G2	mtx1	*MTX1*	Metaxin 1	151952587–151957144	1q22		0.051
G2	c1orf2	*C1orf2*	Chromosome 1 open reading frame 2	151994882–152003120	1q22	ND	ND
G2	hspc003	*MAPBPIP*	Mitogen-activated protein-binding protein-interacting protein	152802478–152806168	1q22	0.076	
G2		*RAB25*	RAB25, member RAS oncogene family	152808855–152818122	1q22		0.001
G2	cct3	*CCT3*	Chaperonin containing TCP1, subunit 3 (gamma)	153056634–153085846	1q22		0.0003
G2	croc4	*C1orf61*	Chromosome 1 open reading frame 61	153128056–153153185	1q22		0.075
							
G3	usf1	*USF1*	Upstream transcription factor 1	157781513–157787199	1q23.3	0.009	0.007
G3	aldh9	*ALDH9A1*	Aldehyde dehydrogenase 9 family, member A1	162327485–162364132	1q24.1	0.088	0.022
G3	mpzl1	*MPZL1*	Myelin protein zero-like 1	164387268–164453994	1q24.2	0.05	0.070
G3	tbx19	*TBX19*	T-box 19	164946309–164979694	1q24.2		0.027
							
G4	plu-1	*JARID1B*	Jumonji, AT rich interactive domain 1B (RBP2-like)	199162987–199245053	1q32.1	0.097	0.095
G4	sox13	*SOX13*	SRY (sex determining region Y)-box 13	200442674–200457500	1q32.1		
G4	pctk3	*PCTK3*	PCTAIRE protein kinase 3	201857380–201862760	1q32.1	0.05	0.001
							
G5	spuf	*NENF*	Neuron-derived neurotrophic factor	209222493–209235935	1q32.3		0.023
G5	esrrg	*ESRRG*	Oestrogen-related receptor gamma	212723109–213309462	1q41		
							
G6	arf1	*ARF1*	ADP-ribosylation factor 1	224655969–224672451	1q42.13	0.098	
G6	rab4	*RAB4A*	RAB4A, member RAS oncogene family	225806272–225839911	1q42.13		0.020
G6	disc1	*DISC1*	Disrupted in schizophrenia 1	228235748–228635487	1q42.2		0.001
G6	tarbp1	*TARBP1*	TAR (HIV) RNA-binding protein 1	230818923–230906713	1q42.2		0.0009
G6	tbce	*TBCE*	Tubulin-specific chaperone e	231749924–231831433	1q42.3		
G6	chs1	*LYST*	Lysosomal trafficking regulator	232120934–232326807	1q42.3		0.010
							
G7	hnrpu	*HNRPU*	Heterogeneous nuclear ribonucleoprotein U	241218474–241229338	1q44	ND	ND

ND=not done and refers to Q–RT–PCR measurements which could not be performed.

RNA expression profiles of 307 genes located at 1q were analysed in a total of 29 breast cancer cell lines and 26 primary tumours. Genes presented correspond to the 30 genes selected by DS. Significance threshold was DS>0.32 corresponding to <0.01 false positive. Expression levels were quantified by Q–RT–PCR for 28 out of 30 genes (primer design was unsuccessful for c1orf2 and *HNRPU*). Quantitative PCR primer sequences are presented in Supplementary Table S2. The recently reported candidate oncogene *RAB25*, which was not present on our array, was quantified as a positive control. Quantitative RT–PCR data were analysed for differential expression using two *t*-test approaches; *t*-test 1 (noted *P*-value 1) indicates correlation with copy number gain; *t*-test 2 (*P*-value 2) differential expression with normal breast. Two significance thresholds were used; strict *P*=<0.05, tolerant *P*=<0.1, *P*-values>0.1 were considered as nonsignificant and only values within the tolerance limit are indicated. Cell lines analysed were: BRCAMZ01, MDAMB175, CAL51, MDAMB435, SKBR7, ZR7530, BT474, MCF7Rich, HS578T, MDAMB436, SUM149, SUM185, BT20, HCC1187, HCC1428, HCC1937, HCC1954, HCC2218, MDAMB157, MDAMB361, MDAMB468, SKBR3, T47D, UACC812 and ZR751.
